# Substrate Stiffness Modulates TGF-β1-Induced Lineage Specification in Multipotent Vascular Stem Cells

**DOI:** 10.3390/cells14080611

**Published:** 2025-04-17

**Authors:** Yujie Yan, Yuhang Wang, Julia S. Chu, Li Yang, Xian Li, Song Li

**Affiliations:** 1 College of Medical Informatics, Chongqing Medical University, Chongqing 400016, China; yanyujie@stu.cqmu.edu.cn (Y.Y.); wangyuhang@stu.cqmu.edu.cn (Y.W.); 2 Department of Neurology, University of California, San Francisco, CA 94143, USA; julia.chu@ucsf.edu; 3 Department of Bioengineering, University of California, Berkeley, CA 94720, USA; 4 Key Laboratory of Biorheological Science and Technology, Ministry of Education, Bioengineering College, Chongqing University, Chongqing 400030, China; yanglibme@cqu.edu.cn; 5 Department of Bioengineering, University of California, Los Angeles, CA 90095, USA; songli@ucla.edu

**Keywords:** multipotent vascular stem cells, substrate stiffness, TGF-β1, smooth muscle cell, chondrogenic

## Abstract

Multipotent vascular stem cells (MVSCs) are found in the vascular wall and surrounding tissues and possess the ability to differentiate into mesenchymal lineages. Previous studies have shown that MVSCs can be activated in response to vascular injury and differentiate into vascular smooth muscle cells (SMCs), contributing to vascular remodeling and microvessel formation. However, it remains unclear as to whether and how microenvironmental changes in the extracellular matrix, such as substrate stiffness, modulates MVSC differentiation under pathological conditions. This study demonstrated that MVSCs cultured on stiff substrates exhibited increased cell spreading, stronger cell adhesion, and a higher expression of SMC markers, including myosin heavy chain (MHC), myocardin (MYCD), calponin 1 (CNN1), and smooth muscle α-actin (SMA). In contrast, MVSCs on soft substrates showed an elevated expression of the chondrogenic markers aggrecan 1 (AGC1) and collagen-II (COL2A1). The presence of TGF-β1 further increased the expression of SMC markers on stiff substrates and chondrogenic markers on the soft substrates. Collectively, these results establish substrate stiffness as a key regulator of MVSC lineage commitment through cytoskeletal reorganization, with TGF-β1 acting as a biochemical amplifier. Our findings highlight the substrate-stiffness-dependent differentiation of MVSCs and provide mechanistic insights into the role of MVSCs in vascular remodeling during atherosclerosis development and blood vessel regeneration.

## 1. Introduction

Vascular diseases such as atherosclerosis (AS) and neointima formation represent significant pathological processes that contribute to cardiovascular morbidity and mortality worldwide. These conditions are characterized by a complex interplay of cellular and molecular events, including endothelial cell dysfunction, inflammatory cell recruitment, and the de-differentiation of vascular smooth muscle cells (SMCs) [1,2,3]. Endothelial dysfunction initiates the disease process by promoting the adhesion and transmigration of inflammatory cells, which in turn release cytokines and growth factors that drive SMC phenotypic switching. This phenotypic transition from a contractile to a synthetic state is a hallmark of vascular remodeling, enabling SMCs to proliferate, migrate, and contribute to neointima formation.

It was observed in our previous studies that multipotent vascular stem cells (MVSCs) in the arterial medial and adventitial layers also contribute to the population of proliferative SMCs in the neointima [4]. These MVSCs, which reside in the vascular wall under normal physiological conditions, remain quiescent. However, under pathological stimuli such as vascular injury or hemodynamic stress, MVSCs were activated and proliferative, and migrated from both the medial and adventitial layers to participate in neointima formation [5]. Despite these advances, the mechanisms by which microenvironmental changes induce MVSC differentiation into synthetic SMCs remain poorly understood.

Biophysical factors, particularly the mechanical properties of the extracellular matrix (ECM), have been widely reported to regulate various cellular functions including adhesion, differentiation, and reprogramming [6,7,8,9,10]. The stiffness of the vascular wall is a dynamic parameter that undergoes significant changes during disease progression. Under normal physiological conditions, arterial stiffness typically ranges from 10 to 40 kPa, providing an optimal mechanical environment for vascular cell function [11,12,13]. However, pathological changes such as fibrosis or calcification can dramatically increase stiffness, while the accumulation of lipids and cholesterol locally reduces stiffness to the 1–20 kPa range, where 1 kPa mimics lipid-rich regions in atherosclerotic plaques and 20 kPa represents early fibrotic transition zones [14]. During AS development, fibrosis and calcification of the vessel wall can further elevate stiffness, often far exceeding normal physiological levels [11,15,16,17]. Despite these insights, it remains to be determined how the mechanical property of extracellular matrix (ECM) regulates MVSC differentiation.

In addition to biophysical cues, biochemical factors such as growth factors also play a critical role in stem cell differentiation and function. Growth factors embedded in the ECM of blood vessels are essential for stimulating adventitial and medial stem cells to differentiate into SMCs [18,19]. Among these, TGF-β1 not only drives stem cell differentiation into SMCs during the development but also regulates SMC phenotype during vascular remodeling [20]. Furthermore, TGF-β1 has been shown to promote the specification of other types of stem cells, such as bone marrow mesenchymal stem cells (MSCs) and neural crest stem cells (NCSCs) into SMCs. It enhances the expression of contractile SMC markers, including calponin 1 (CNN1), smooth muscle α-actin (SMA), and myosin heavy chain (MHC) [20]. Given the dual influence of mechanical and biochemical cues, we sought to investigate how substrate stiffness, in combination with TGF-β1, regulates MVSC differentiation into SMCs.

Here, we utilized polyacrylamide (PA) gels and collagen gels to modulate the stiffness of the ECM, creating substrates that mimic the mechanical properties of both healthy and diseased vascular tissues [14,21]. Through comprehensive gene profiling and cell differentiation analysis, we investigated the effects of substrate stiffness and TGF-β1 on MVSC differentiation. Our findings reveal that the interplay between mechanical and biochemical cues significantly influences the fate of MVSCs, providing new insights into the roles of MVSCs in vascular remodeling.

## 2. Materials and Methods

### 2.1. Cell Isolation, Culture, and Characterization

Animal experiments in this study were approved by the Institutional Animal Care and Use Committee of Chongqing Medical University (No. IACUC-CQMU-2024-0879). Multipotent vascular stem cells (MVSCs) were isolated from the tunica media of arteries throughout the bodies of Sprague–Dawley (SD) rats using the enzymatic digestion method, as described previously [3]. Briefly, tissues were incubated with 3 mg/mL of type II collagenase (Sigma-Aldrich, St. Louis, MO, USA) in DMEM with a 1/5 (*w*/*v*) ratio of tissue (g) to enzyme solution (mL). After incubation at 37 °C for 30 min, the same volume of 1 mg/mL elastase (Sigma-Aldrich, St. Louis, MO, USA) solution was added to the solution containing the tissue and collagenase. The tissues were incubated for another 1–2 h until all of the tissues were digested. Cells were then seeded onto dishes pre-coated with 1% CellStart (Thermo Fisher Scientific, Carlsbad, CA, USA) and maintained at 37 °C and 5% CO_2_ in DMEM with 2% chick embryo extract (MP Biomedical, Santa Ana, CA, USA), 1% FBS (Thermo Fisher Scientific, Carlsbad, CA, USA), 1% N2 (Thermo Fisher Scientific, Carlsbad, CA, USA), 2% B27 (Thermo Fisher Scientific, Carlsbad, CA, USA), 100 nM retinoic acid (Sigma-Aldrich, St. Louis, MO, USA), 50 nM 2-mercaptoethanol (Sigma-Aldrich, St. Louis, MO, USA), 1% penicillin/streptomycin, and 20 ng/mL bFGF (R&D Systems, Minneapolis, MN, USA) (maintenance medium).

The expanded MVSCs were characterized by the expression of neural crest markers (SOX10, SOX17). MVSC differentiation into neural lineages (Schwann cells, peripheral neurons) and mesenchymal lineages (SMCs, adipocytes, osteoblasts, and chondrocytes) was performed and confirmed using the protocol described previously [22].

All experiments were conducted using MVSCs at 70–80% confluency. Unless specified, cells were maintained in DMEM supplemented with 10% FBS and 1% penicillin/streptomycin. To evaluate the structural and functional changes in cells, cells were analyzed after 1 day of culture to capture initial cytoskeletal responses to substrate stiffness; differentiation markers were assessed on cells cultured for 3 days (immunofluorescence and microarray) or 7 days (qPCR). In differentiation studies, the culture medium was supplemented with 10 ng/mL TGF-β1 (PeproTech, Rocky Hill, NJ, USA).

### 2.2. Fabrication of Substrates with Various Stiffnesses

The first approach involved preparing stiff substrates and polyacrylamide (PA) gels with a Young’s moduli of 20 kPa and 1 kPa by modulating the acrylamide/bis-acrylamide ratios according to established protocols [14]. The fabrication process consisted of the following steps: First, glass slides were functionalized with 3-aminopropyltrimethoxysilane and 0.5% glutaraldehyde. For PA gel preparation, acrylamide and bis-acrylamide solutions at predetermined concentrations were polymerized to form 200 µm thick gels on the slides. by our recent atomic force microscopy (AFM) analysis [14]; these fabrication methods produce substrates with highly consistent mechanical properties, demonstrating less than 5% variation in the Young’s modulus over 7-day culture periods. The gel surfaces were then conjugated with rat tail collagen-I (10 µg/cm^2^, BD BioSciences, San Jose, CA, USA) using Sulfo-SANPAH as a crosslinker. Collagen-I solution (1 mg/mL, pH 7.4) was prepared by diluting 4 mg/mL of rat-tail-collagen stock solution and neutralizing with NaOH prior to polymerization. A standardized protocol was carried out to ensure collagen density consistency, as described previously [20]. Stiff substrates were prepared by directly coating the glass slides with collagen-I at the same surface density. As was confirmed prior to cell seeding, all substrates were sterilized by UV treatment.

For the studies using collagen gel, 500 µm thick collagen-I gels were prepared by polymerizing 1 mL of neutralized collagen-I solution per well in 24-well plates at 37 °C for 1 h. Based on the published characterization of this concentration and polymerization protocol, these gels exhibit a Young’s modulus of <1 kPa [23,24]. For stiff substrates, unpolymerized collagen-I solution (same concentration as that for the collagen gel) was used to rinse the culture dishes, and excess solution was removed, resulting in a thin-layer (micrometers) coating of polymerized collagen.

### 2.3. RNA Isolation for Oligonucleotide Microarray and qPCR

MVSCs isolated from the carotid arteries of SD rats were lysed using Trizol reagent for total RNA extraction, following established protocols [3]. For microarray analysis, the purified RNA was resuspended in nuclease-free water and adjusted to a final concentration of 0.5 mg/mL. Aliquots (10 µL) of each sample were then subjected to genome-wide expression profiling using an Affymetrix GeneChip U133AA of the Av2 array platform, which contains 31,099 probe sets. Sample labeling and hybridization were performed in strict accordance with the manufacturer’s recommended protocols. Signal intensities were obtained for all probe sets and organized using GeneTraffic microarray analysis software (version 3.2). For differentially expressed genes (DEGs) between different cell types, the limma package in R (version 4.3.1) was used to analyze DEGs between different cell types. Heatmaps and dot plots were generated by R.

For qPCR, RNA pellets were resuspended in DEPC-treated H_2_O. The cDNA synthesis was performed using the ThermoScript RT-PCR System (Thermo Fisher Scientific, Carlsbad, CA, USA) in a two-step reverse transcription process. Quantitative PCR was then carried out using SYBR Green reagent (Thermo Fisher Scientific, Carlsbad, CA, USA) on an ABI Prism 7000 Sequence Detection System (Thermo Fisher Scientific, Foster City, CA, USA). Gene-specific primer sequences are provided in Table 1. Gene expression levels were normalized to endogenous 18S ribosomal RNA content in the corresponding samples.

### 2.4. Staining and Histological Analysis

For immunofluorescence staining, MVSCs were fixed with 4% paraformaldehyde (PFA) in PBS for 15 min, permeabilized with 0.5% Triton X-100 (Sigma-Aldrich, St. Louis, MO, USA), and blocked with 1% BSA (Sigma-Aldrich, St. Louis, MO, USA). Actin cytoskeleton staining was performed by incubating samples with FITC-conjugated phalloidin (Thermo Fisher Scientific, Carlsbad, CA, USA) for 30 min to visualize filamentous actin (F-actin), while other cell markers were detected by incubating samples with specific primary antibodies for 2 h at room temperature, followed by three PBS washes and subsequent incubation with appropriate Alexa-488- and/or Alexa-546-labelled secondary antibodies (Thermo Fisher Scientific, Carlsbad, CA, USA). Finally, nuclei were counterstained with 4,6-diamidino-2-phenylindole (DAPI; Thermo Fisher Scientific, Carlsbad, CA, USA) to display blue fluorescence. Fluorescence imaging was performed using a Zeiss LSM710 confocal microscope system, equipped with He/Ne laser sources and a Zeiss Axio Observer microscope. Z-section images (0.3–0.5 μm thickness) were acquired and projected into a single composite image for analysis. All images within an experimental group were captured using identical hardware configurations and software settings. The settings of the microscopy system such as the exposure time were maintained consistently throughout all experiments.

The following primary antibodies were used in the study including SOX10 (R&D Systems, Minneapolis, MN, USA), SOX17 (R&D Systems, Minneapolis, MN, USA), GFAP (Millipore, Billerica, MA, USA), S100β (Sigma-Aldrich, St. Louis, MO, USA), Tuj1 (Covance, Princeton, NJ, USA), Peripherin (Millipore, Billerica, MA, USA), SMA (Sigma-Aldrich, St. Louis, MO, USA), CNN1 (Epitomics, Burlingame, CA, USA), MHC (Santa Cruz, Biotechnology, Dallas, TX, USA), alkaline phosphatase (ALP; DSHB, Iowa city, IA, USA), collagen-II (Col-II; Millipore, Billerica, MA, USA), phalloidin (Thermo Fisher Scientific, Carlsbad, CA, USA), and vinculin (Sigma-Aldrich, St. Louis, MO, USA).

To quantify actin cytoskeleton organization and focal adhesion distribution, phalloidin and vinculin staining was analyzed using ImageJ (v1.54, NIH). Two parameters were measured: (1) the cell spreading area (μm^2^), determined by the fluorescence area analysis in the phalloidin-stained images, with a threshold adjustment to measure the positive fluorescence area; (2) the number of focal adhesions per cell, analyzed using the focal adhesion analysis macro in ImageJ to automatically identify vinculin-positive focal adhesions (≥0.5 μm^2^), followed by manual counting with ImageJ’s built-in counting tool [25,26]. Three independent experiments were performed, with ≥30 cells analyzed per condition.

For organic dye staining, cells or the tissue sections were fixed with 4% PFA for 30 min, washed, and stained with Oil Red O (Sigma-Aldrich, St. Louis, MO, USA), Alizarin Red S (Sigma-Aldrich, St. Louis, MO, USA), or Alcian Blue (Sigma-Aldrich, St. Louis, MO, USA) according to the manufacturers’ instructions. Images were collected by a Zeiss Axioskop 2 plus microscope (Oberkochen, Germany).

### 2.5. Statistical Analysis

To account for variations in the baseline between experiments, individual sample values were normalized to their corresponding controls, after which the normalized ratios from all experiments were pooled and subjected to statistical analysis using log-transformed t-tests (microarray: *p* < 0.05, n = 4; qPCR: *p* < 0.05, n = 3). For comparisons between two specific conditions, two-tailed t-tests were employed (qPCR: *p* < 0.05, n = 3), while multiple comparisons were assessed using either one-way or two-way ANOVA, as appropriate, with all experimental data expressed as the mean ± SD.

## 3. Results

### 3.1. Characterization and Multipotency of MVSCs

**Figure 1:**
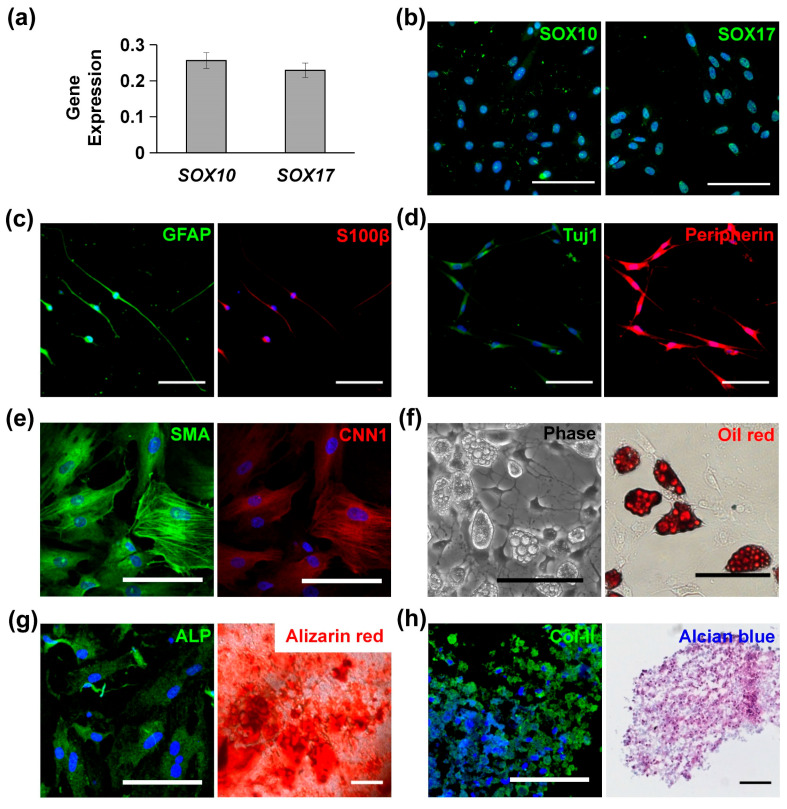
Characterization and differentiation potential of multipotent vascular stem cells (MVSCs): (**a**) qPCR and (**b**) immunofluorescence staining of MVSC markers (*SOX10*, *SOX17*). (**c**) Immunofluorescence staining for Schwann markers GFAP and S100β. (**d**) Immunofluorescence staining for peripheral neuron markers Tuj1 and peripherin. (**e**) Immunofluorescence staining for smooth muscle cell (SMC) markers SMA and CNN1. (**f**) Adipocytes for lipid droplets using phase contrast imaging and Oil Red O staining. (**g**) Osteogenic differentiation was shown by immunofluorescence staining of ALP and Alizarin Red S staining for calcified matrix. (**h**) Chondrogenic differentiation was shown by immunofluorescence staining of Col-II and Alcian Blue staining for glycosaminoglycans. In all immunofluorescence images, nuclei were stained by DAPI in blue. Scale bars = 100 µm.

MVSCs isolated from rat arteries were characterized with high levels of *SOX10* and *SOX17* expression, as confirmed by qPCR (Figure 1a) and immunofluorescence staining (Figure 1b). When cultured in specific induction media, MVSCs demonstrated the ability to differentiate into Schwann cells (Figure 1c), peripheral neurons (Figure 1d), SMCs (Figure 1e), adipogenic cells (Figure 1f), osteoblastic cells (Figure 1g), and chondrogenic cells (Figure 1h). These results confirmed the multipotent differentiation potential of MVSCs.

### 3.2. Varying the Stiffness of PA Gels Modulates Cytoskeleton Organization

**Figure 2:**
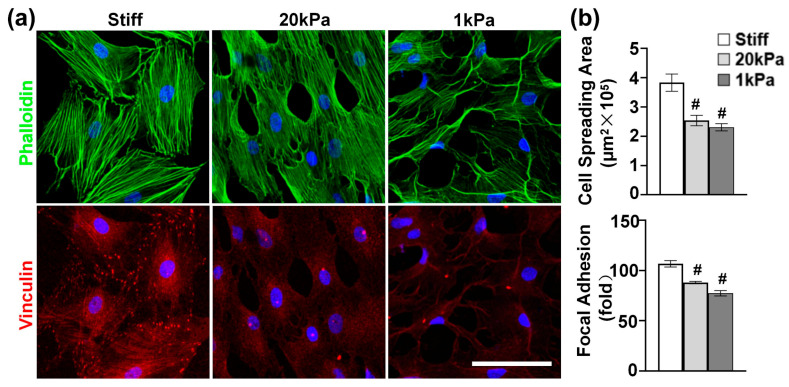
(**a**) Effects of varying stiffnesses on actin cytoskeleton of MVSCs. MVSCs were cultured on stiff substrates or PA gels (20 kPa and 1 kPa) for 1 day and stained for F-actin (phalloidin, green) and focal adhesion (vinculin, red). Nuclei were stained by DAPI in blue. Scale bars = 100 µm. (**b**) Quantitative analysis of cell spreading area and the number of focal adhesions per cell. # indicates a significant difference from data in MVSCs on stiff substrates (*p* < 0.05).

Given the critical role of the actin cytoskeleton in sensing and transducing extracellular biophysical signals to modulate cell functions [27], we investigated the effect of stiffness on actin cytoskeleton organization. MVSCs were cultured on stiff substrates or PA gels (20 kPa and 1 kPa) for 1 day and stained for F-actin and focal adhesions. According to Figure 2a, the MVSCs on stiff substrates exhibited extensive stress fibers with well-organized vinculin-rich focal adhesions predominantly localized at the stress fiber termini, indicating mature adhesion complexes that mediate strong mechanical coupling between the extracellular matrix and cytoskeleton. In contrast, the cells on 20 kPa and 1 kPa PA gels showed a reduced cell spreading area by 33.8% and 39.8%, respectively (Figure 2b); fewer organized stress fibers; smaller and more diffuse vinculin puncta; and increased filopodia formation with peripheral vinculin accumulation. Specifically, the number of focal adhesions per cell was reduced by 17.6% on 20 kPa PA gels and by 28.0% on 1 kPa PA gels, compared to those on stiff substrates. These results demonstrate that substrate stiffness directly regulates focal adhesion assembly, which in turn influences cytoskeletal architecture and cellular tension distribution.

### 3.3. Microarray Analysis of MVSCs on PA Gels with Varying Stiffnesses

**Figure 3:**
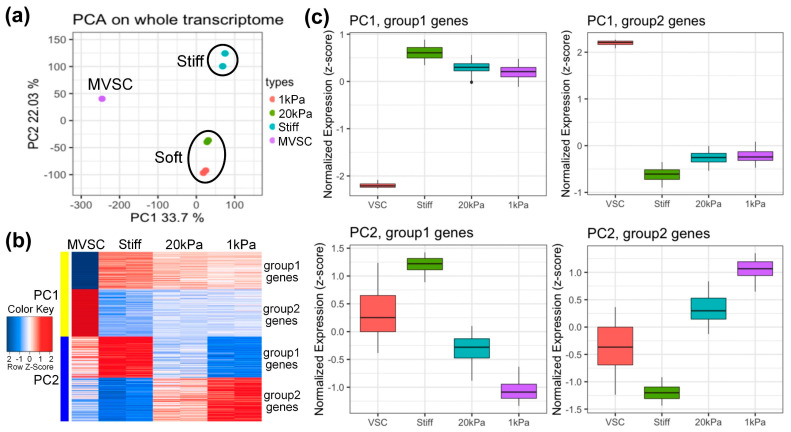
Microarray analysis of MVSCs on varying stiffnesses. (**a**) Principal component analysis (PCA) on whole transcriptome. The first principal component (PC1, *X*-axis) separated the stem cell/differentiated cells; the second principal component (PC2, *Y*-axis) separated cells on stiff or soft substrates. (**b**) Heatmap and (**c**) boxplot analysis of top 500 genes on the correlation with PC1 and PC2. MVSC: MVSCs in maintenance medium as a control. Stiff, 20 kPa and 1 kPa: MVSCs on stiff substrates or PA gels (20 kPa and 1 kPa).

To explore gene expression profiles, MVSCs were seeded on stiff substrates or PA gels (20 kPa and 1 kPa) for 3 days, with the MVSCs in a maintenance medium serving as a control. RNA samples were then subjected to microarray analysis. Log-scale scatter plots of signal intensity values from all 31,099 probe sets from each set of microarray hybridizations were analyzed for statistical significance and sorted by fold change (relative to the MVSC control). As is shown in Figure 3a, the principal component analysis (PCA) plot reveals the similarity of each sample in terms of their whole transcriptome profiles. The first principal component (PC1, *X*-axis) separated stem cells from differentiated cells, while the second principal component (PC2, *Y*-axis) distinguished cells cultured on soft or stiff substrates. The data indicated that independent samples cultured on soft surfaces (20 kPa and 1 kPa) clustered together, suggesting similar transcriptome profiles.

We extracted the top 500 genes from PC1 and PC2 for further analysis. Heatmaps and boxplots (Figure 3b,c) showed that these genes were divided into four groups. PC1 group 1 genes were highly expressed in differentiated cells, while group 2 genes were highly expressed in stem cells. For PC2, group 1 genes were highly expressed in stiff substrates, and group 2 genes were highly expressed in soft substrates (1 kDa).

**Figure 4:**
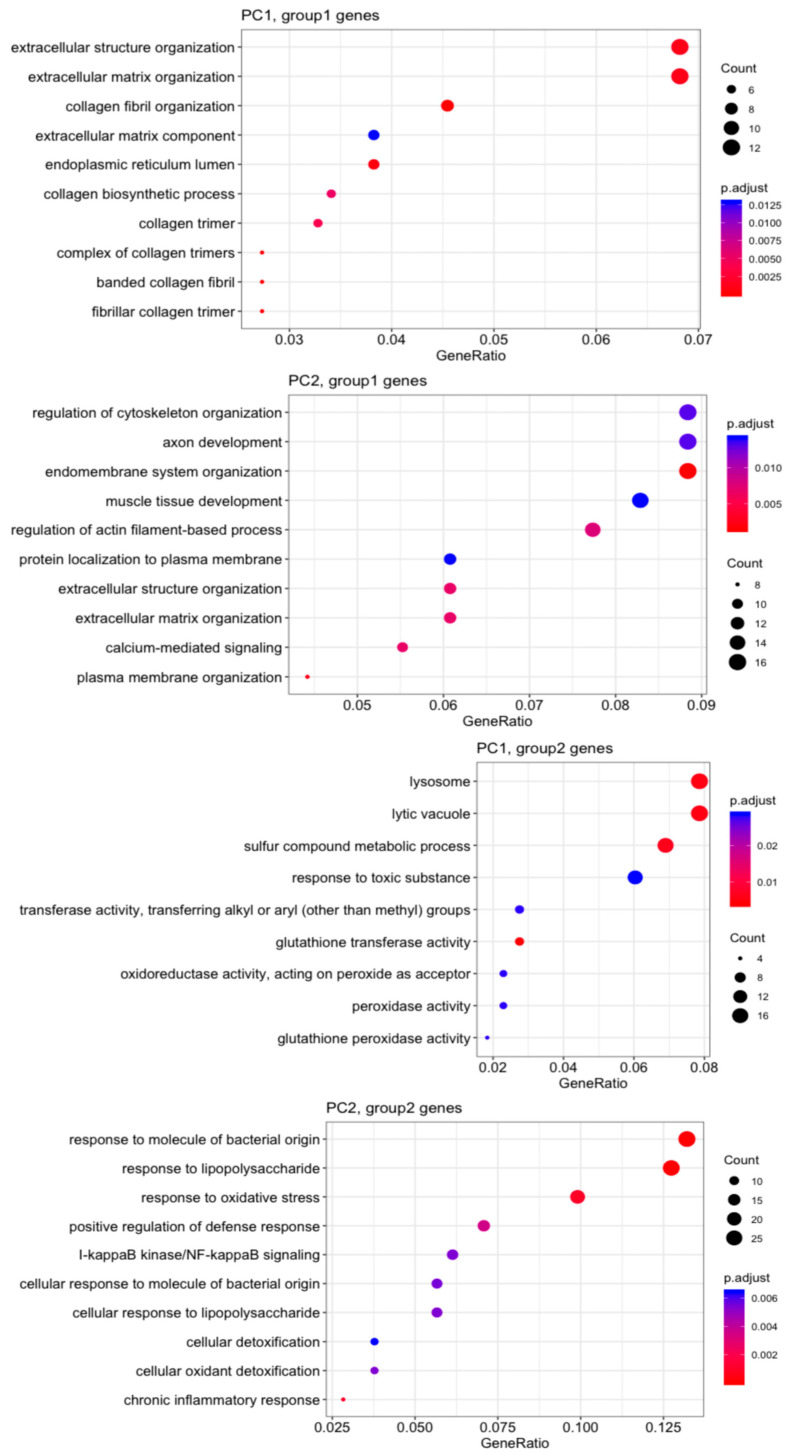
Gene Ontology (GO) analysis of top 500 genes on the correlation with PC1 and PC2.

Gene Ontology (GO) analysis (Figure 4) revealed that MVSCs cultured on stiff substrates or PA gels (20 kPa and 1 kPa) upregulated genes related to the ECM and structure organization (PC1, group1 genes), but downregulated lysosome-related genes (PC1, group2 genes) compared to the MVSC control. Additionally, stiff substrates promoted genes associated with endomembrane function and muscle tissue development, which were not evident in the 20 kPa or 1 kPa samples (PC2, group1 genes). In contrast, the soft surfaces (20 kPa and 1 kPa) enhanced lipopolysaccharide-associated genes (PC2, group2 genes), potentially indicating an inflammatory response.

**Figure 5:**
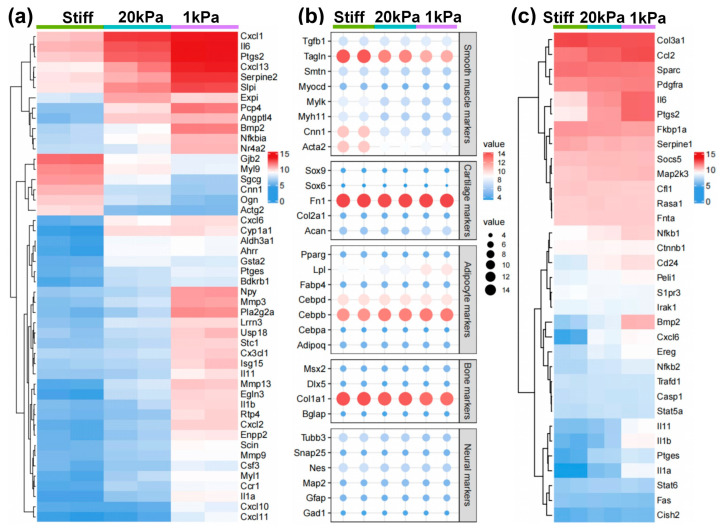
Heatmap showing the expression of specific lineage-related genes and proinflammatory cytokines. (**a**) Heatmap showing the differentially expressed genes (DEGs) between different cell types. Genes were selected based on a fold-change cutoff of >3 (*|logFC*| > 3) and a *p*-value cutoff of <0.05 (*p* < 0.05). (**b**) Dot plots showing the expression of specific lineage-related genes, including markers for SMC, chondrogenic, adipogenic, osteogenic, and neural lineages. (**c**) Heatmap illustrating the proinflammatory cytokines in different cell types.

Genes showing significant differential expression were further analyzed based on the substrate stiffness, as shown in Figure 5. The heatmaps and dot plots illustrate that the increase in stiffness enhanced SMC-related genes, while the decrease in stiffness slightly induced chondrogenic lineage markers (Figure 5a,b). Meanwhile, proinflammatory cytokines were highly expressed with the decrease in stiffness (Figure 5c). In contrast, other lineage-specific markers including those for adipogenic, osteogenic, and neural lineages, showed no significant change among various stiffnesses (Figure 5b). These results suggest that stiff substrates promote MVSC transition into the SMC lineage, while soft substrates tend to induce inflammation and chondrogenic differentiation.

### 3.4. Stiff Substrates Promote MVSC Differentiation into SMC Lineage

**Figure 6:**
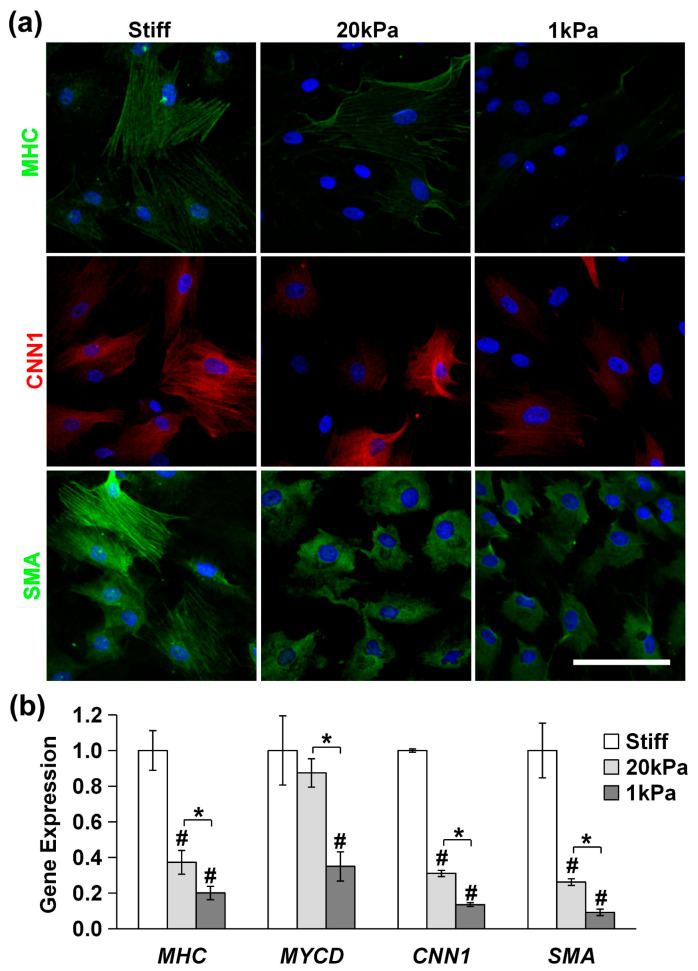
Effects of substrate stiffness on MVSC differentiation into SMCs on PA gels. MVSCs were cultured on stiff substrates or PA gels (20 kPa and 1 kPa) for 3 or 7 days, followed by immunofluorescence staining or qPCR analysis, respectively. (**a**) Immunofluorescence images of MVSCs for SMC markers including MHC, CNN1, and SMA. Nuclei were stained by DAPI in blue. Scale bars = 100 µm. (**b**) Gene expression of SMC markers including *MHC*, *MYCD*, *CNN1*, and *SMA*. * indicates a significant difference (*p* < 0.05). # indicates a significant difference compared to gene expression in MVSCs on stiff substrates (*p* < 0.05).

We used immunofluorescence staining and qPCR to assess the expression of differentiation markers. As is shown in Figure 6a, the expression of the SMC markers MHC, CNN1, and SMA in MVSCs decreased rapidly with the decrease in matrix rigidity (stiff, 20 kPa and 1 kPa). Similarly, the qPCR results in Figure 6b revealed that at 20 kPa, the expression of *MHC* decreased by 63%, *MYCD* by 14%, *CNN1* by 69%, and *SMA* by 74%. At 1 kPa, the expression of *MHC* decreased by 80%, *MYCD* by 65%, *CNN1* by 86%, and *SMA* by 91%. These results indicate that stiff substrates significantly enhance the differentiation of MVSCs into the SMC lineage.

### 3.5. Validation of Cytoskeleton Organization and Lineage Commitment of MVSCs on Varying Stiffnesses of Collagen Gels and TGF-β1

**Figure 7:**
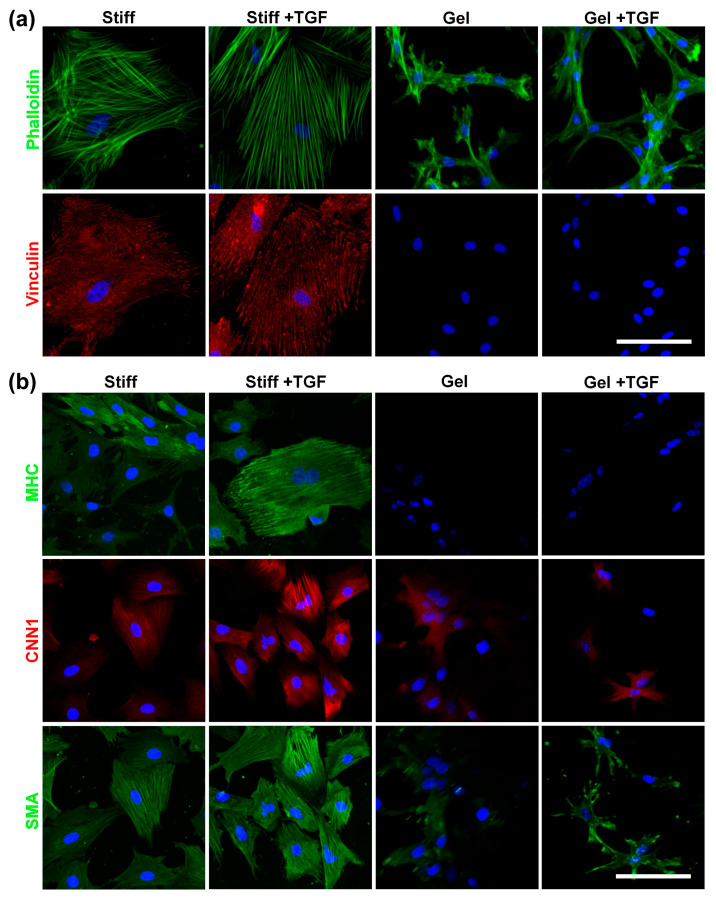
Effects of varying stiffnesses on actin cytoskeleton organization and MVSC differentiation into SMCs on collagen gels with TGF-β1 treatment. MVSCs were cultured on stiff substrates or collagen gels and treated with TGF-β1 for 1 or 3 days, followed by immunofluorescence staining. (**a**) Double staining of F-actin (phalloidin, green) and focal adhesion (vinculin, red) in MVSCs after 1 day of cell culture. (**b**) Immunofluorescence images of MVSCs for SMC markers including MHC, CNN1, and SMA after 3 days of culture. Nuclei were stained by DAPI in blue. Scale bars = 100 µm.

To investigate the combined effects of substrate stiffness and TGF-β1, we used collagen-I-coated stiff substrates and collagen gels (soft substrate) as representatives of a wide range of stiffnesses. MVSCs were seeded onto these surfaces in the absence or presence of TGF-β1, and the effects of stiffness on cell morphology and differentiation were assessed. As is shown in Figure 7a, cells on stiffer substrates exhibited greater spreading, more stress fibers, and increased focal adhesion formation. In addition, TGF-β1 induced thicker stress fibers in MVSCs on stiff substrates but not on collagen gels. Vinculin staining of focal adhesions mirrored the trend observed for actin filaments.

**Figure 8:**
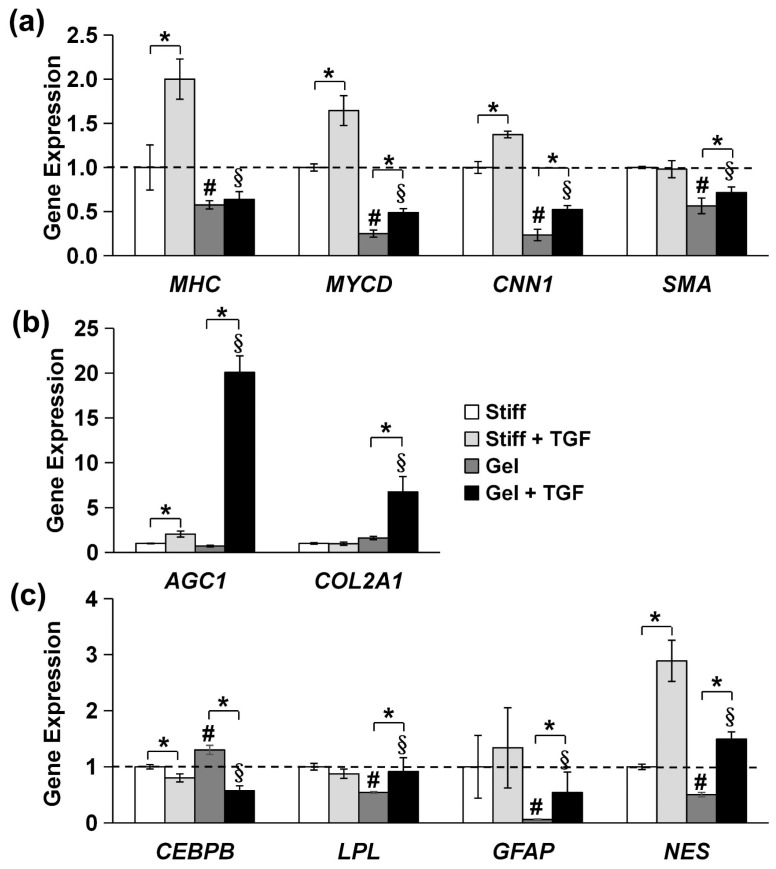
Effects of substrate stiffness on gene expression of specific lineage-related markers in MVSCs treated with TGF-β1. MVSCs were cultured on stiff substrates or collagen gels and treated with TGF-β1 for 3 days, followed by qPCR analysis, showing gene expression of (**a**) SMC markers (*MHC*, *MYCD*, *CNN1* and *SMA*), (**b**) chondrogenic markers (*AGC1*, *COL2A1*), (**c**) adipogenic markers (*CEBPB*, *LPL*), and neural markers (*GFAP*, *NES*). * indicates a significant difference (*p* < 0.05); # indicates a significant difference compared to gene expression in MVSCs on stiff substrates (*p* < 0.05); § indicates a significant difference compared to gene expression in MVSCs on stiff substrates treated with TGF-β1 (*p* < 0.05).

We further validated the tendency of MVSCs to differentiate into the SMC lineage using immunofluorescence staining and qPCR. As is shown in Figure 7b, MVSCs displayed higher expression levels of SMC markers on stiff substrates compared to collagen gels, consistent with our previous observation on PA gels (Figure 6). Moreover, the addition of TGF-β1 significantly induced the expression of SMC markers in MVSCs on stiff substrates but had minimal effects on collagen gels. Specifically, as is shown by the qPCR results in Figure 8a, *MHC* expression levels in MVSCs seeded on stiff substrates containing TGF-β1 were 2-fold higher than those on stiff substrates without TGF-β1 and 3.5-fold higher than those on collagen gels. Similarly, *MYCD* expression levels were 1.6-fold higher on stiff substrates with TGF-β1 compared to stiff substrates without TGF-β1 and 6.6-fold higher than those on collagen gels. *CNN1* expression levels were 1.4-fold higher on stiff substrates with TGF-β1 compared to stiff substrates without TGF-β1 and 5.9-fold higher than those on collagen gels. *SMA* expression levels were approximately 1.7-fold higher on stiff substrates with TGF-β1 compared to collagen gels. These results suggest that MVSCs on stiff substrates exhibit greater differentiation into the SMC lineage compared to those on collagen gels, and TGF-β1 further amplifies this effect on stiff substrates, while its impact is diminished on soft substrates.

In addition, the chondrogenic markers *AGC1* and *COL2A1* were significantly upregulated in MVSCs cultured on collagen gels compared to those on stiff substrates in the presence of TGF-β1 (Figure 8b), with a 9.9-fold increase in *AGC1* and a 6.9-fold increase in *COL2A1*. These striking differences indicate that soft substrates and TGF-β1 promote lineage specification toward the chondrogenic lineage.

Lastly, we examined the expression of other specific lineage-related markers, including adipogenic markers (*CEBPB* and *LPL*) and neural markers (*GFAP* and *Nestin*). The qPCR analysis revealed no significant changes in the expression of these genes between stiff and soft substrates of collagen gels in terms of the decrease in substrate stiffness (Figure 8c). Although *Nestin* expression was elevated in the presence of TGF-β1, the trend was inconsistent with the expression of the other neural marker *GFAP*. These results suggest that substrate stiffness has minimal effects on adipogenic or neural lineage commitment. Instead, substrate stiffness primarily regulates the differentiation of MVSCs into SMC or chondrogenic lineages.

## 4. Discussion

This study offers a comprehensive exploration of the multipotency of MVSCs and the role of substrate stiffness in regulating cytoskeleton organization and differentiation. Our findings reveal that MVSCs isolated from rat arteries exhibit high expression levels of SOX10 and SOX17, confirming their stem-cell-like properties. These cells demonstrate the ability to differentiate into multiple lineages, including Schwann cells, peripheral neurons, SMCs, adipocytes, osteoblasts, and chondrocytes, highlighting their multipotent nature. This aligns with the results of previous studies on vascular stem cells, which have shown a similar differentiation potential in response to specific induction media [28].

MVSCs in the arterial wall may play a significant role in vascular diseases and remodeling [22,29,30]. During vascular injury, MVSCs became proliferative and may differentiate into SMCs, contributing to vascular remodeling and neointimal formation [3]. This differentiation potential extends beyond injury responses; in vascular regeneration, MVSCs can be recruited to the outer surface of vascular grafts, where they differentiate into SMCs to facilitate graft integration and vascular remodeling [31]. Similarly, in wound healing and scar formation, MVSCs in the soft tissues surrounding blood vessels can differentiate into both myofibroblasts and SMCs, contributing to biomaterial encapsulation and microvascularization in vivo [22]. Lineage tracing studies have further provided compelling evidence that MVSCs are implicated in vascular fibrosis and the formation of intimal lesions following injury [32,33]. Notably, the chondrogenic potential of MVSCs may also contribute to late-stage vascular pathologies such as atherosclerosis, where cartilage-like structures are observed in calcified plaques. This suggests that MVSC-derived chondrogenesis could play a role in disease progression, particularly in the transition from soft, lipid-rich lesions to calcified, cartilage-like plaques. Collectively, these findings highlight the critical role of MVSC expansion and differentiation in vascular pathology, including fibrosis, remodeling, and disease progression.

The actin cytoskeleton plays a critical role in sensing and transducing extracellular biophysical signals, which modulate cell behavior [21,34,35]. Our results indicate that substrate stiffness significantly influences the cytoskeletal organization of MVSCs. Specifically, we found that stiffness-induced cytoskeletal rearrangements directly correlate with lineage-specific differentiation. Cells cultured on stiff substrates exhibited extensive stress fibers and well-defined focal adhesions, whereas those on softer PA gels showed reduced spreading, fewer stress fibers, and increased filopodia formation. These observations are consistent with previous studies demonstrating that substrate stiffness modulates cytoskeletal dynamics, with stiffer substrates promoting stress fiber formation and cell spreading [7,36]. The reduced stress fibers and increased filopodia on softer substrates suggest that MVSCs adopt a more exploratory phenotype in response to lower mechanical resistance, which may facilitate their adaptation to softer microenvironments. This cytoskeletal plasticity likely serves as a mechanical switch, directing cells toward stiffness-specific differentiation.

Microarray analysis revealed distinct gene expression profiles in MVSCs cultured on substrates of varying stiffnesses. Principal component analysis (PCA) demonstrated that PC1 separated stem cells from differentiated cells, while PC2 distinguished cells cultured on stiff versus soft substrates. Notably, cells cultured on soft substrates (20 kPa and 1 kPa) clustered together, indicating similar transcriptomic profiles. This suggests that substrate stiffness is a key determinant of MVSC gene expression, with softer substrates promoting a more stem-cell-like state. GO analysis further highlighted that stiff substrates upregulated genes related to the ECM organization and muscle tissue development, while downregulating lysosome-related genes. In contrast, soft substrates enhanced the expression of lipopolysaccharide-associated genes, potentially indicating an inflammatory response. These findings are consistent with previous studies showing that stiff substrates promote SMC differentiation, while softer substrates favor chondrogenic differentiation [20].

Our results demonstrate that substrate stiffness plays a pivotal role in directing MVSC lineage commitment. Notably, the stiffness-dependent cytoskeletal rearrangements precede and predict differentiation outcomes, suggesting a causal relationship. Stiff substrates significantly enhanced the expression of SMC markers, while softer substrates slightly induced chondrogenic markers. This stiffness-dependent differentiation was further validated using collagen gels, where MVSCs on stiff substrates exhibited the higher expression of SMC markers compared to those on soft substrates. The addition of TGF-β1, a known inducer of SMC differentiation, further amplified this effect on stiff substrates but had a minimal impact on soft substrates. These findings suggest that the mechanical properties of the substrate synergize with biochemical cues to regulate MVSC differentiation. Interestingly, soft substrates in combination with TGF-β1 promoted chondrogenic differentiation, as evidenced by the upregulation of chondrogenic markers. This is consistent with previous studies showing that soft substrates favor chondrogenic lineage commitment, likely due to the reduced mechanical resistance that mimics the native cartilage microenvironment [37]. In contrast, adipogenic and neural lineage markers showed no significant changes in response to substrate stiffness, indicating that MVSC differentiation into these lineages is less sensitive to mechanical cues.

The influence of substrate stiffness on cell differentiation extends beyond MVSCs to other multipotent stem cells capable of differentiating into mesenchymal lineages. Our previous in vitro studies have demonstrated that stiff substrates promote the differentiation of MSCs or NCSCs into SMCs, whereas soft substrates drive these cells toward chondrogenic or glial lineages, respectively [20]. These differentiation processes are further amplified in the presence of TGF-β1, highlighting the synergistic effects of mechanical and biochemical signals. In addition, the differentiation of NCSCs into SMCs in vascular grafts has been shown to be stiffness-dependent, reinforcing the importance of substrate stiffness in regulating stem cell fate. These findings highlight the critical role of mechanical cues in stem cell biology and their potential applications in tissue engineering [38].

The findings from this study have significant implications for tissue engineering and regenerative medicine. The ability of MVSCs to differentiate into multiple lineages in response to substrate stiffness highlights their potential for use in cell-based therapies for vascular and musculoskeletal disorders. For instance, the promotion of SMC differentiation on stiff substrates could be leveraged for vascular tissue engineering, where the mechanical properties of the scaffold can be tailored to mimic the native vascular environment [39]. Conversely, the induction of chondrogenic differentiation on soft substrates could be utilized for cartilage repair strategies.

Moreover, the observed inflammatory response on soft substrates warrants further investigation. While the upregulation of lipopolysaccharide-associated genes may indicate an inflammatory phenotype, it is unclear whether this response is beneficial or detrimental in the context of tissue regeneration [40,41]. Future studies should explore the functional consequences of this inflammatory response and its potential impact on MVSC differentiation and tissue repair.

While our study demonstrates the significant role of substrate stiffness in directing MVSC fate, we recognize several important methodological considerations regarding substrate selection. Although glass is not physiological, stiff substrates based on collagen-I-coated glass (70 GPa, serving as the stiff control for PA experiments) and tissue-culture plastic (~3 GPa, as the stiff control for collagen hydrogel studies) were used as a standardized stiff-surface control in this study, which was consistent with established mechanobiology protocols [14,20]. While we acknowledge that these substrates exceed the physiological stiffness ranges and lack the viscoelastic properties of native tissues, our control experiments showed no significant difference in chondrogenic markers between glass and tissue-culture plastic (Supplemental Figure S1). This consistency suggests that the observed chondrogenic preference on soft substrates reflects a stiffness-dependent response rather than material-specific effects, particularly given that all substrates were uniformly coated with collagen-I to minimize surface chemistry variations. Collectively, these findings imply that an extreme stiffness contrast (kPa vs. GPa) plays a predominant role in lineage specification under these controlled conditions. It should be noted that physiological microenvironments exhibit viscoelastic properties not captured by these rigid controls. Thus, while our study clarifies the impact of stiffness extremes, further work is needed to refine the mechanical threshold by investigating intermediate stiffness ranges and evaluating the independent contributions of stiffness and viscoelasticity across physiological ranges.

In conclusion, this study demonstrates that MVSCs possess multipotent differentiation potential and that substrate stiffness is a critical regulator of their lineage commitment. Our data establish that stiffness-induced cytoskeletal rearrangement is a mechanism by which mechanical cues guide MVSC differentiation into SMCs. Stiff substrates promote SMC differentiation, while soft substrates favor chondrogenic differentiation and may induce an inflammatory response. These findings underscore the importance of considering both mechanical and biochemical cues in the design of biomaterials for tissue engineering applications. Future research should focus on elucidating the molecular mechanisms underlying stiffness-dependent differentiation and exploring the therapeutic potential of MVSCs in regenerative medicine. Additionally, the role of MVSCs in vascular diseases, such as atherosclerosis (AS), warrants further investigation, as these cells may contribute to fibrotic, chondrogenic, and osteogenic remodeling during disease progression. Understanding the regulatory mechanisms of MVSC differentiation in response to mechanical and biochemical cues could provide new insights into vascular pathology and inform the development of targeted therapies.

## 5. Conclusions

In this study, we demonstrated that substrate stiffness, together with TGF-β1, regulate the differentiation of MVSCs into SMCs or chondrocytes in a stiffness-dependent manner. These findings provide insights into the mechanisms of vascular remodeling during disease development, and may lead to therapeutic strategies targeting MVSCs and ECM components. Moreover, multipotent MVSCs can be used to engineer vascular tissues for regeneration and disease modeling.

## Figures and Tables

**Table 1:** Primers for qPCR.

Gene Name	Forward Primer (5′ to 3′)	Reverse Primer (5′ to 3′)
SOX10	CTGGAGGTTGCTGAACGAGAGT	GTCCGGATGGTCTTTTTTGTG
SOX17	AGAACCCGGATCTGCACAAC	AGGATTTGCCTAGCATCTTGCT
CNN1	AGAACAAGCTGGCCCAGAAA	CACCCCTTCGATCCACTCTCT
SMA	TCCTGACCCTGAAGTATCCGATA	GGTGCCAGATCTTTTCCATGTC
MHC	TTCCGGCAACGCTACGA	TCCATCCATGAAGCCTTTGG
MYCD	TCTTACAGTTACGGCTTCAACAGAGA	CCTCGGGTCATGGAACTCA
AGC1	CTTCAAGCTGAACTATGACCACTTTACT	CATGGTCTGGAACTTCTTCTGAGA
COL2A1	CCAGGGCTCCAATGATGTG	GTGTTTCGTGCAGCCATCCT
CEBPB	CGCCTTTAGACCCATGGAAGT	AGGCAGTCGGGCTCGTAGTAG
LPL	TCTCTTGGGATACAGCCTTGGA	GCCAGTAATTCTGTTGACTTTCTTATTG

## Data Availability

Data presented in this study are contained within this article are available upon request to the corresponding author.

## Supplementary Materials

The following supporting information can be downloaded at: https://www.mdpi.com/article/10.3390/cells14080611/s1, Figure S1: Comparative analysis of chondrogenic marker COL2A1 expression in MVSCs cultured on different stiff substrates.

## Abbreviations

The following abbreviations are used in this manuscript:

AS	Atherosclerosis
MVSCs	Multipotent vascular stem cells
SMCs	Vascular smooth muscle cells
MHC	Myosin heavy chain
MYCD	Myocardin
CNN1	Calponin 1
SMA	Smooth muscle α-actin
ECM	Extracellular matrix
PA	Polyacrylamide
MSCs	Bone marrow mesenchymal stem cells
NCSCs	Neural crest stem cells
DEGs	Differentially expressed genes
AGC1	Aggrecan 1
COL2A1	Collagen-II
CEBPB	CCAAT/enhancer binding protein beta
LPL	Lipoprotein lipase
PFA	Paraformaldehyde
F-actin	Filamentous actin
DAPI	4,6-diamidino-2-phenylindole
ALP	Alkaline phosphatase
PCA	Principal component analysis
PC	Principal component
GO	Gene Ontology

## References

[1] Ross R. (1999). Atherosclerosis—An inflammatory disease. N. Engl. J. Med..

[2] Libby P., Hansson G.K. (2015). Inflammation and immunity in diseases of the arterial tree: Players and layers. Circ. Res..

[3] Tang Z., Wang A., Yuan F., Yan Z., Liu B., Chu J.S., Helms J.A., Li S. (2012). Differentiation of multipotent vascular stem cells contributes to vascular diseases. Nat. Commun..

[4] Yuan F.L., Wang D., Xu K., Wang J.X., Zhang Z.J., Yang L., Yang G.Y., Li S. (2017). Contribution of vascular cells to neointimal formation. PLoS ONE.

[5] Ciavarella C., Valente S., Pasquinelli G. (2022). The The characteristics and survival potential under sub-lethal stress of mesenchymal stromal/stem cells isolated from the human vascular wall. Stem Cells.

[6] Putra V.D.L., Kilian K.A., Knothe Tate M.L. (2023). Biomechanical, biophysical and biochemical modulators of cytoskeletal remodelling and emergent stem cell lineage commitment. Commun. Biol..

[7] Engler A.J., Sen S., Sweeney H.L., Discher D.E. (2006). Matrix elasticity directs stem cell lineage specification. Cell.

[8] Barcena A.J.R., Mishra A., Bolinas D.K.M., Martin B.M., Melancon M.P. (2024). Integration of electrospun scaffolds and biological polymers for enhancing the delivery and efficacy of mesen-chymal stem/stromal cell therapies. Front. Biosci..

[9] Kureel S.K., Maroto R., Davis K., Sheetz M. (2025). Cellular mechanical memory: A potential tool for mesenchymal stem cell-based therapy. Stem Cell Res. Ther..

[10] Saraswathibhatla A., Indana D., Chaudhuri O. (2023). Cell-extracellular matrix mechanotransduction in 3d. Nat. Rev. Mol. Cell Biol..

[11] Lyle A.N., Raaz U. (2017). Killing me unsoftly: Causes and mechanisms of arterial stiffness. Arterioscler. Thromb. Vasc. Biol..

[12] Huynh J., Nishimura N., Rana K., Peloquin J.M., Califano J.P., Montague C.R., King M.R., Schaffer C.B., Reinhart-King C.A. (2011). Age-related intimal stiffening enhances endothelial permeability and leukocyte transmigration. Sci. Transl. Med..

[13] Qiu H., Zhu Y., Sun Z., Trzeciakowski J.P., Gansner M., Depre C., Resuello R.R., Natividad F.F., Hunter W.C., Genin G.M. (2010). Short communication: Vascular smooth muscle cell stiffness as a mechanism for increased aortic stiffness with aging. Circ. Res..

[14] Song Y., Soto J., Wong S.Y., Wu Y., Hoffman T., Akhtar N., Norris S., Chu J., Park H., Kelkhoff D.O. (2024). Biphasic regulation of epigenetic state by matrix stiffness during cell reprogramming. Sci. Adv..

[15] Matsumoto T., Abe H., Ohashi T., Kato Y., Sato M. (2002). Local elastic modulus of atherosclerotic lesions of rabbit thoracic aortas measured by pipette aspiration method. Physiol. Meas..

[16] Hansen L., Taylor W.R. (2016). Is increased arterial stiffness a cause or consequence of atherosclerosis?. Atherosclerosis.

[17] Raaz U., Zollner A.M., Schellinger I.N., Toh R., Nakagami F., Brandt M., Emrich F.C., Kayama Y., Eken S., Adam M. (2015). Segmental aortic stiffening contributes to experimental abdominal aortic aneurysm development. Circulation.

[18] Sainz J., Al Haj Zen A., Caligiuri G., Demerens C., Urbain D., Lemitre M., Lafont A. (2006). Isolation of “side population” progenitor cells from healthy arteries of adult mice. Arterioscler. Thromb. Vasc. Biol..

[19] Vissers G., Giacomozzi M., Verdurmen W., Peek R., Nap A. (2024). The role of fibrosis in endometriosis: A systematic review. Hum. Reprod. Update.

[20] Park J.S., Chu J.S., Tsou A.D., Diop R., Tang Z., Wang A., Li S. (2011). The effect of matrix stiffness on the differentiation of mesenchymal stem cells in response to tgf-beta. Biomaterials.

[21] Cao H., Duan L., Zhang Y., Cao J., Zhang K. (2021). Current hydrogel advances in physicochemical and biological response-driven biomedical application diversity. Signal Transduct. Target. Ther..

[22] Wang D., Wang A., Wu F., Qiu X., Li Y., Chu J., Huang W.C., Xu K., Gong X., Li S. (2017). Sox10^+^ adult stem cells contribute to biomaterial encapsulation and microvascularization. Sci. Rep..

[23] Li S., Lao J., Chen B.P., Li Y.S., Zhao Y., Chu J., Chen K.D., Tsou T.C., Peck K., Chien S. (2003). Genomic analysis of smooth muscle cells in 3-dimensional collagen matrix. FASEB J..

[24] Roeder B.A., Kokini K., Sturgis J.E., Robinson J.P., Voytik-Harbin S.L. (2002). Tensile mechanical properties of three-dimensional type i collagen extracellular matrices with varied microstructure. J. Biomech. Eng..

[25] Pan M., Chew T.W., Wong D.C.P., Xiao J., Ong H.T., Chin J.F.L., Low B.C. (2020). Bnip-2 retards breast cancer cell migration by coupling microtubule-mediated gef-h1 and RhoA activation. Sci. Adv..

[26] Sun A.R., Ramli M.F.H., Shen X., Chen D., Foo R.S., Zhu J., Ackers-Johnson M., Young J.L. (2023). Hybrid hydrogel-extracellular matrix scaffolds identify distinct ligand and mechanical signatures in cardiac aging. bioRxiv.

[27] Vining K.H., Mooney D.J. (2017). Mechanical forces direct stem cell behaviour in development and regeneration. Nat. Rev. Mol. Cell Biol..

[28] Majesky M.W., Dong X.R., Regan J.N., Hoglund V.J. (2011). Vascular smooth muscle progenitor cells: Building and repairing blood vessels. Circ. Res..

[29] Han Y., Yan L., Xia L., Li S., Zhang Q., Jin C. (2022). Global trends and frontier topics about vascular smooth muscle cells phenotype switch: A bibliometric analysis from 1999 to 2021. Front. Pharmacol..

[30] Du L., Sun X., Gong H., Wang T., Jiang L., Huang C., Xu X., Li Z., Xu H., Ma L. (2023). Single cell and lineage tracing studies reveal the impact of CD34^+^ cells on myocardial fibrosis during heart failure. Stem Cell Res. Ther..

[31] Lyu L., Li Z., Wen Z., He Y., Wang X., Jiang L., Zhou X., Huang C., Wu Y., Chen T. (2023). Fate mapping rna-sequencing reveal malat1 regulates sca1^+^ progenitor cells to vascular smooth muscle cells transition in vascular remodeling. Cell Mol. Life Sci..

[32] Di Luca M., Fitzpatrick E., Burtenshaw D., Liu W., Helt J.-C., Hakimjavadi R., Corcoran E., Gusti Y., Sheridan D., Harman S. (2021). The calcium binding protein s100β marks hedgehog-responsive resident vascular stem cells within vascular lesions. Npj Regen. Med..

[33] Molony C., King D., Di Luca M., Kitching M., Olayinka A., Hakimjavadi R., Julius L.A.N., Fitzpatrick E., Gusti Y., Burtenshaw D. (2021). Disease-relevant single cell photonic signatures identify s100β stem cells and their myogenic progeny in vascu-lar lesions. Stem Cell Rev. Rep..

[34] Kanchanawong P., Calderwood D.A. (2023). Organization, dynamics and mechanoregulation of integrin-mediated cell-ecm adhesions. Nat. Rev. Mol. Cell Biol..

[35] Seetharaman S., Etienne-Manneville S. (2020). Cytoskeletal crosstalk in cell migration. Trends Cell Biol..

[36] Discher D.E., Janmey P., Wang Y.L. (2005). Tissue cells feel and respond to the stiffness of their substrate. Science.

[37] Huey D.J., Hu J.C., Athanasiou K.A. (2012). Unlike bone, cartilage regeneration remains elusive. Science.

[38] Yi B., Xu Q., Liu W. (2021). An overview of substrate stiffness guided cellular response and its applications in tissue regeneration. Bioact. Mater..

[39] Lang Z., Chen T., Zhu S., Wu X., Wu Y., Miao X., Wang Q., Zhao L., Zhu Z., Xu R.X. (2024). Construction of vascular grafts based on tissue-engineered scaffolds. Mater. Today Bio.

[40] Hyun J., Kim S.J., Cho S.D., Kim H.W. (2023). Mechano-modulation of T cells for cancer immunotherapy. Biomaterials.

[41] Lacolley P., Regnault V., Segers P., Laurent S. (2017). Vascular smooth muscle cells and arterial stiffening: Relevance in development, aging, and disease. Physiol. Rev..

