# Supplementary material for: Substrate Stiffness Modulates TGF-β1-Induced Lineage Specification in Multipotent Vascular Stem Cells
Source: Cells. 2025 Apr 17;14(8):611. doi: 10.3390/cells14080611 (PMC12025518; doi:10.3390/cells14080611)
Supplement: Supplementary file 1 [file cells-14-00611-s001.zip › Figure S1.pptx]

## Slide 1
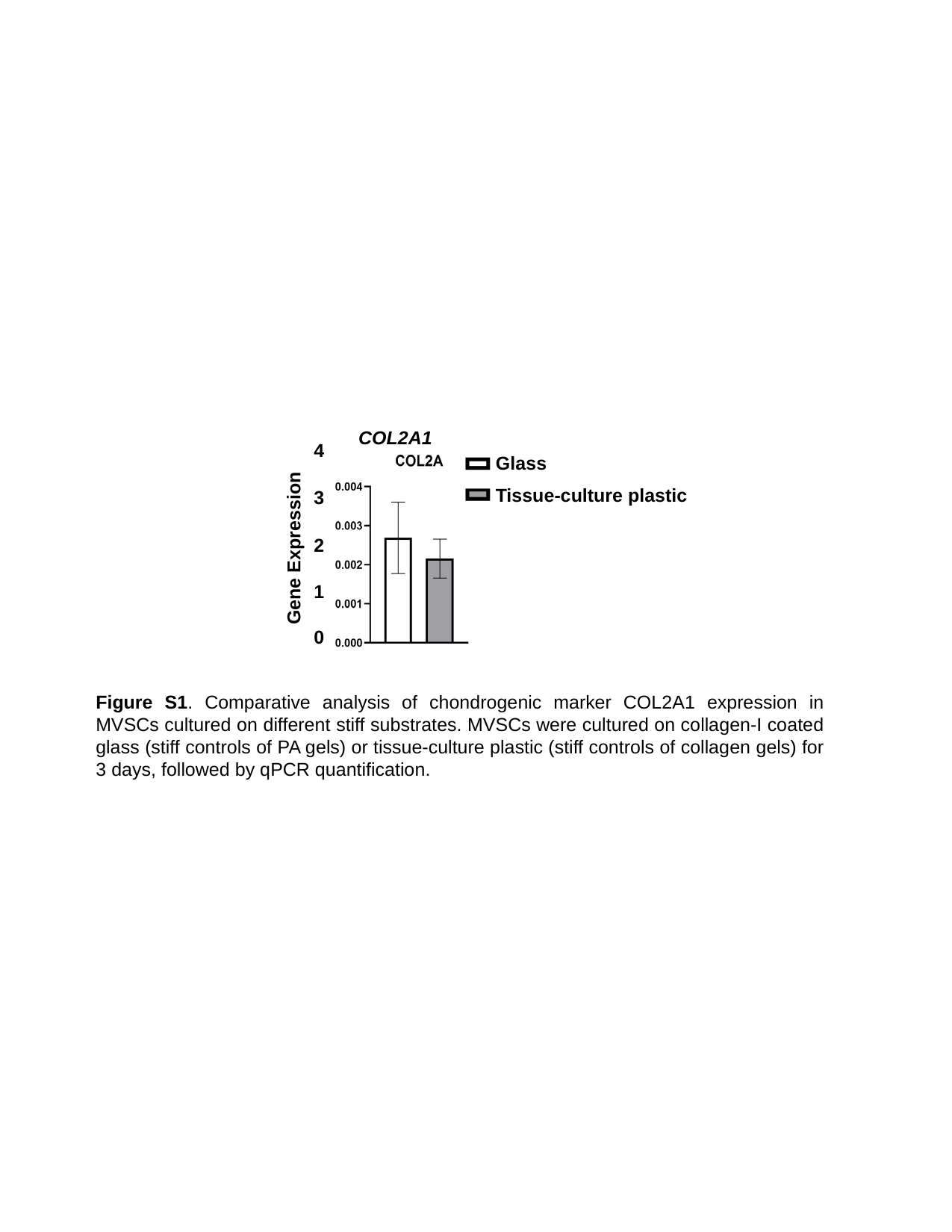

COL2A1
4
Glass
Gene Expression
Tissue-culture plastic
3
2
1
0
Figure S1. Comparative analysis of chondrogenic marker COL2A1 expression in MVSCs cultured on different stiff substrates. MVSCs were cultured on collagen-I coated glass (stiff controls of PA gels) or tissue-culture plastic (stiff controls of collagen gels) for 3 days, followed by qPCR quantification.
